# Ex vivo evaluation of a multilayered sealant patch for watertight dural closure: cranial and spinal models

**DOI:** 10.1007/s10856-021-06552-4

**Published:** 2021-07-23

**Authors:** A. Kinaci, S. van Thoor, S. Redegeld, M. Tooren, T. P. C. van Doormaal

**Affiliations:** 1 Department of Neurology and Neurosurgery, Brain Center Rudolph Magnus, University Medical Centre Utrecht, Utrecht, The Netherlands; 2 Brain Technology Institute, Utrecht, The Netherlands; 3 Polyganics BV, Groningen, The Netherlands

## Abstract

Cerebrospinal fluid leakage is a frequent complication after cranial and spinal surgery. To prevent this complication and seal the dura watertight, we developed Liqoseal, a dural sealant patch comprising a watertight polyesterurethane layer and an adhesive layer consisting of poly(DL-lactide-co-ε-caprolactone) copolymer and multiarmed N-hydroxylsuccinimide functionalized polyethylene glycol. We compared acute burst pressure and resistance to physiological conditions for 72 h of Liqoseal, Adherus, Duraseal, Tachosil, and Tisseel using computer-assisted models and fresh porcine dura. The mean acute burst pressure of Liqoseal in the cranial model (145 ± 39 mmHg) was higher than that of Adherus (87 ± 47 mmHg), Duraseal (51 ± 42 mmHg) and Tachosil (71 ± 16 mmHg). Under physiological conditions, cranial model resistance test results showed that 2 of 3 Liqoseal sealants maintained dural attachment during 72 hours as opposed to 3 of 3 for Adherus and Duraseal and 0 of 3 for Tachosil. The mean burst pressure of Liqoseal in the spinal model (233 ± 81 mmHg) was higher than that of Tachosil (123 ± 63 mmHg) and Tisseel (23 ± 16 mmHg). Under physiological conditions, spinal model resistance test results showed that 2 of 3 Liqoseal sealants maintained dural attachment for 72 hours as opposed to 3 of 3 for Adherus and 0 of 3 for Duraseal and Tachosil. This novel study showed that Liqoseal is capable of achieving a strong watertight seal over a dural defect in ex vivo models.

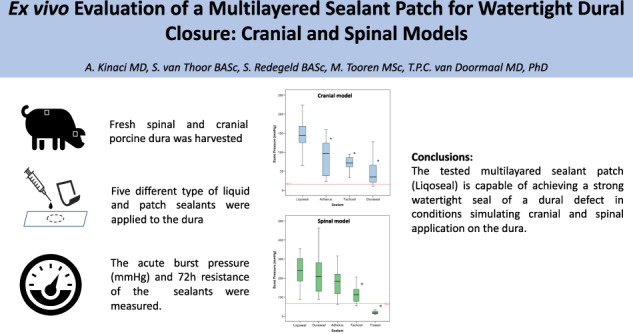

## Introduction

Cerebrospinal fluid (CSF) leakage is a potentially severe complication in neurosurgery that occurs in 8.2–13.8% of all cases following intradural surgery or because of an unintended durotomy in extradural spinal surgery [1, 2].

**Fig. 1:**
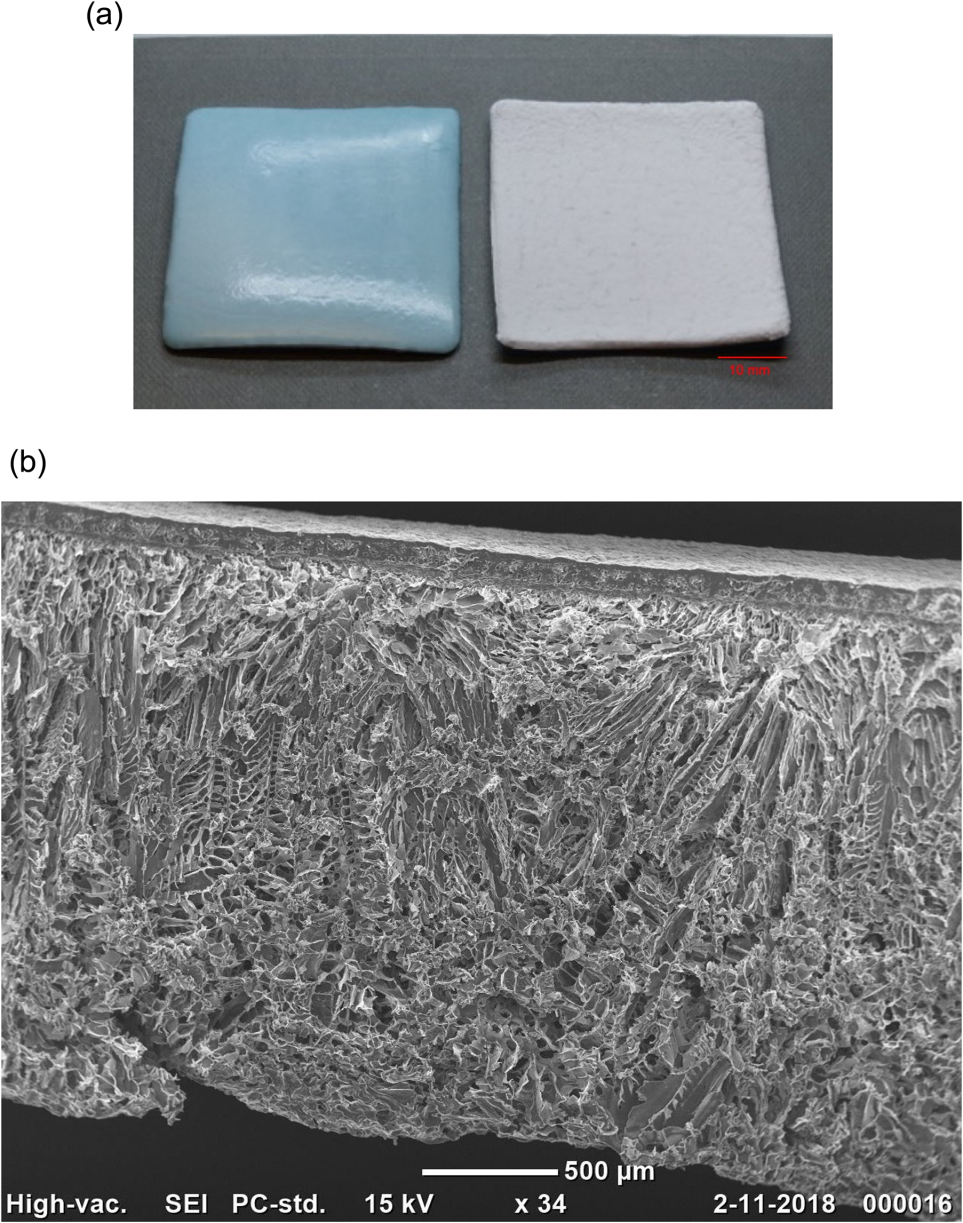
**a** Liqoseal (40 × 40 mm), consisting of a nonadhesive watertight blue layer and an adhesive white layer. **b** A scanning electron microscopy image of Liqoseal showing the multilayered material

Persistent leakage of CSF can lead to secondary complications, such as postural headache, durocutaneous fistula, impaired wound healing, wound infection, meningitis, and intracranial hemorrhage [3–6]. Watertight closure of the dura is considered a cornerstone of treatment to prevent CSF leakage. However, true watertight primary closure through suturing is thought to be impossible as leakage can occur even through a stitch hole [7, 8]. Sealants are used to achieve watertight dural closure after primary closure or in combination with dural substitutes [9]. However, recent systematic reviews have shown that clinically used sealants do not reduce the rate of CSF leakage after cranial and spinal surgery [1, 2]. An ex vivo model was designed to test the effectiveness of the most clinically used sealants in an objective and standardized manner [10]. This model showed that most of the current sealants failed to resist physiological intracranial pressure (16 mmHg) or that the sealants detached from the dura under chronic physiological conditions. Therefore, there is a need for an effective dural sealant to prevent CSF leakage. Hetero, a new multilayered dural sealant patch named Liqoseal (Polyganics B.V., Groningen, The Netherlands), has been developed for watertight closure of the dura. A patch sealant was developed rather than a liquid sealant, as patch sealants do not require preparation time during surgery, they have no mixture inconsistencies, and are of uniform thickness. Moreover, an applicator is not required for patch sealants, which can clog or harbor other issues such as unequal dosing. The sealant consists of two layers, namely, a watertight layer comprising biodegradable polyesterurethane (PU) and an adhesive layer comprising biodegradable poly(DL-lactide-co-ε-caprolactone) copolymer (DL-PLCL) and multiarmed N-hydroxylsuccinimide (NHS) functionalized polyethylene glycol (PEG-NHS) (Fig. 1).

The nonadhesive, upper (blue) PU layer gives the sealant its mechanical properties and is watertight. We synthesized PU from an aliphatic butanediisocyanate that eliminates toxicity and shows excellent biocompatibility [11]. Degradation of butanediisocyanate forms butanediamines that are present in mammalian cells and can ultimately be excreted via urine [12]. Incorporating hydrolytically degradable ester bonds, DL-lactide and ε-caprolactone formed from the monomers resulted in a biodegradable polymer [11, 13].

The lower, white adhesive part of the Liqoseal consists of an amorphous polyester copolymer (DL-PLCL). This material has previously been successfully used as biomaterial in nerve reconstruction surgery [14–16]. DL-PLCL undergoes hydrolytic degradation through random scission of ester bonds. The monomers degrade ultimately into water, caproic acid, and lactic acid [17]. In vitro and in vivo experiments have shown 80% degradation of DL-PLCL in 22 weeks and a very mild foreign body reaction without cytotoxicity and hemolysis [18]. The PEG-NHS in the DL-PLCL is non-immunogenic and nontoxic and degrades through hydrolysis of ester bonds, which have minimal site-to-site and patient-to-patient variation [19–23]. The PEG-NHS adheres to the dura when NHS-ester groups come into contact with amines present in dura. Combining PU with DL-PLCL gives a Liqoseal that is pliable, watertight, and flexible to follow the curvature of the dura. Moreover, PU does not contain adhesive material and ensures that Liqoseal has only one side with adhesive properties. The expected complete degradation period of Liqoseal, based on the biodegradation profile of the polymers, is 1 year [24]. Liqoseal is stored at −18 °C and should be thawed 10 min before application.

We aimed to evaluate Liqoseal using the same cranial model in which the currently used dural sealants had been tested, and to compare the performance of Liqoseal with the current sealants. Subsequently, we adjusted the cranial setup for spinal use, since spinal and cranial characteristics differ. Liqoseal and the currently used dural sealants were studied using this spinal model. The results of Liqoseal were compared with those of the currently used dural sealants. This is the first study who evaluated the performance of Liqoseal in an ex vivo model and the performance of the current sealants in a spinal model

## Materials and methods

### Part I: ex vivo cranial model

#### Acute burst pressure test

**Fig. 2:**
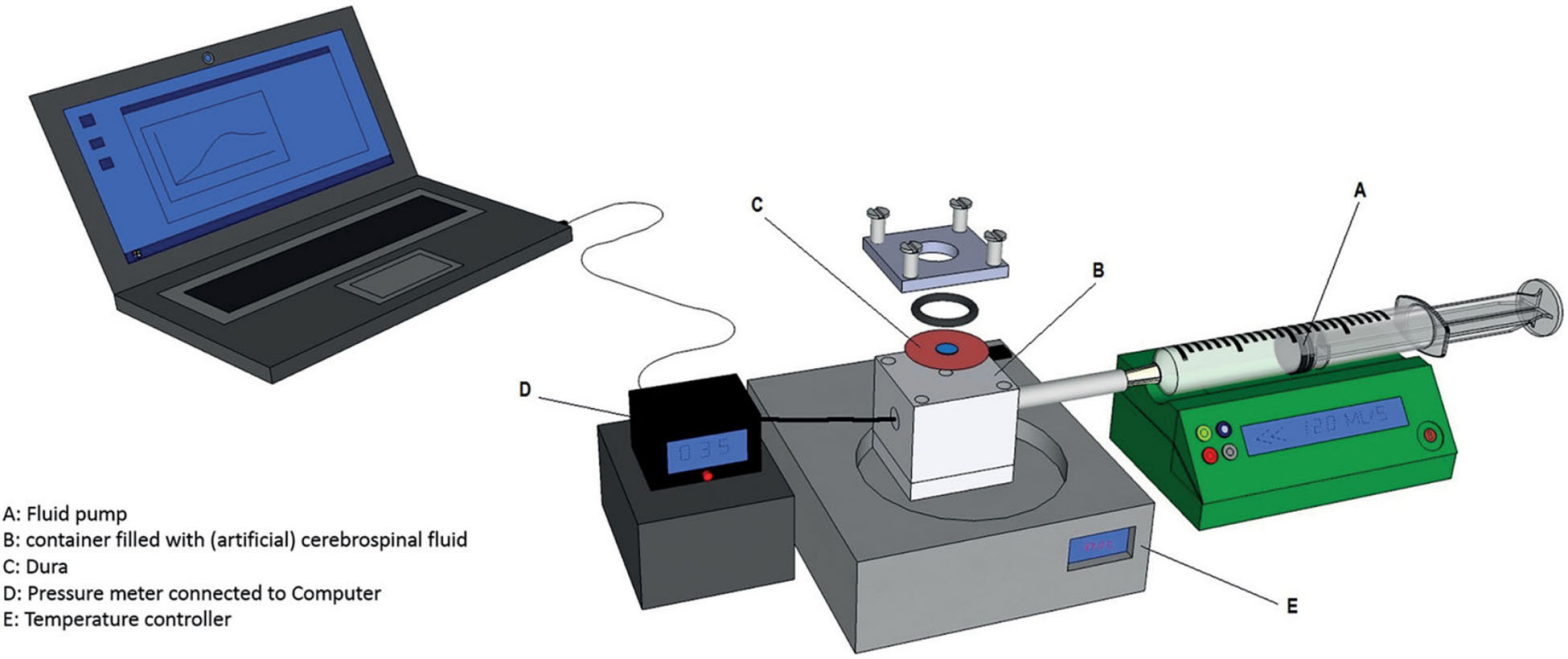
Ex vivo cranial acute burst pressure setup [adapted with permission from ref. [10]]

We used a cranial ex vivo model from a prior study to benchmark the new sealant for cranial application. The design of the ex vivo setups have been described in a previous article [10]. In short, dura mater from Dutch Landrace pigs was obtained at an abattoir and cut into circles of 30 mm. Subsequently, a 3-mm circular gap was punched out from the middle with a dedicated perforator. After thawing, Liqoseal was cut into a circle of 15 mm and applied to the dura covering the gap with 2 min compression. As no information was available in the literature about compression weight, we determined the weight through letting 3 neurosurgeons compress a weight scale imitating sealant application in a real patient. This resulted in a mean weight of 1000 g. A standardized weight was used to exclude compression variance, which may affect adhesion strength. For the acute burst pressure test, the dura with Liqoseal was then clamped above an open pressure chamber, creating a watertight closure. The pressure in the chamber was gradually increased via a fluid pump providing a constant flow of 2.0 mL/min artificial CSF (EcoCyte Bioscience, Germany). A computer with Dasylab software (Dasylab v. 11.0, Norton, USA) continuously measured pressures via a probe and determined maximum pressure (burst pressure) (Fig. 2). The acute burst pressure results of Liqoseal were compared with the acute burst pressure results of the clinically used sealants, with a mean burst pressure above physiological intracranial pressure [10]. The following clinically used sealants were included:

(i) Adherus^®^ Autospray Extended Tip Dural Sealant (Stryker Corporation, Michigan, USA),
(ii) DuraSeal^®^ Xact Sealant System (Integra LifeSciences Corporation, New Jersey, USA)
(iii) TachoSil^®^ Sealant Matrix (Takeda Pharmaceutical Company, Tokyo, Japan).

Adherus is a liquid sealant comprised of two components. To prepare the sealant, the two precursor solutions were first mixed within the supplied applicator, which resulted in crosslinking and formation of a hydrogel [25]. Duraseal is a liquid sealant comprised of two components. The two components were mixed during application in the supplied syringe, which resulted in crosslinking and formation of a hydrogel [26]. Tachosil is a hemostatic collagen fleece-bound sealant patch comprising human fibrinogen and thrombin and equine collagen [6], which can be applied directly onto tissue [6].

#### Cranial resistance test

**Fig. 3:**
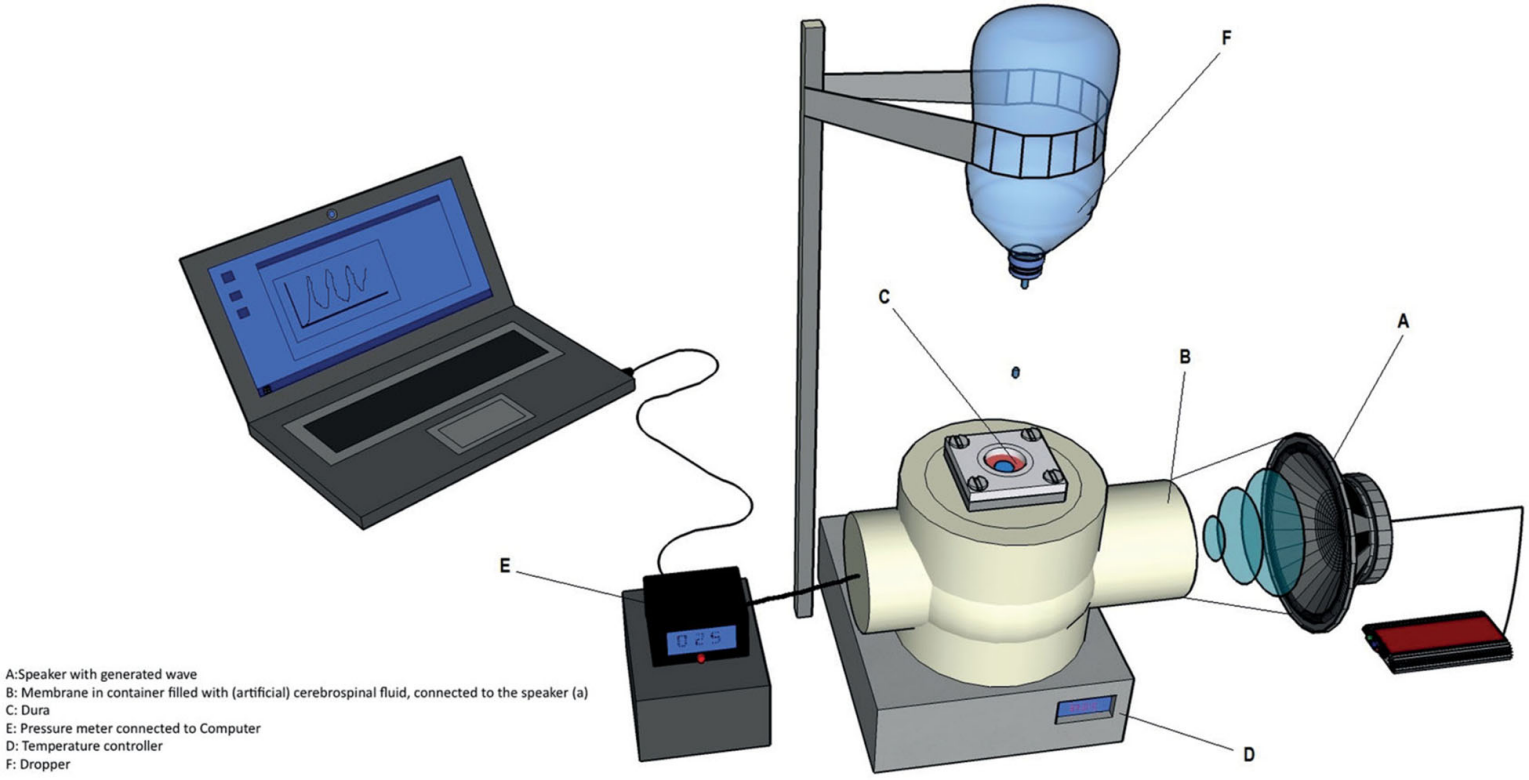
Ex vivo cranial resistance setup [adapted with permission from ref. [10]]

For the resistance test, Liqoseal sealant was exposed to physiological intracranial conditions for 72 h in the same model as described previously [10]. In short, a pressure chamber with a membrane wall was connected to a speaker. This speaker generated a waveform and created pressure on the dura to mimic physiologic intracranial pressure between 6 and 16 mmHg. The dura with sealant was prepared and clamped in the setup, as described in the acute burst pressure test. The temperature of the pressure chamber was maintained at 37 °C. Saline was dripped on top of the dura and sealant at a constant speed to prevent drying. A probe was used to continuously register the pressure in the chamber (Fig. 3). Any leakage of CSF was characterized as a kink in the pressure-time graph. Data were compared with resistance test results of the clinically used Adherus, Duraseal, and Tachosil sealants, evaluated using the same model.

### Part II: ex vivo spinal model

#### Acute burst pressure test

**Fig. 4:**
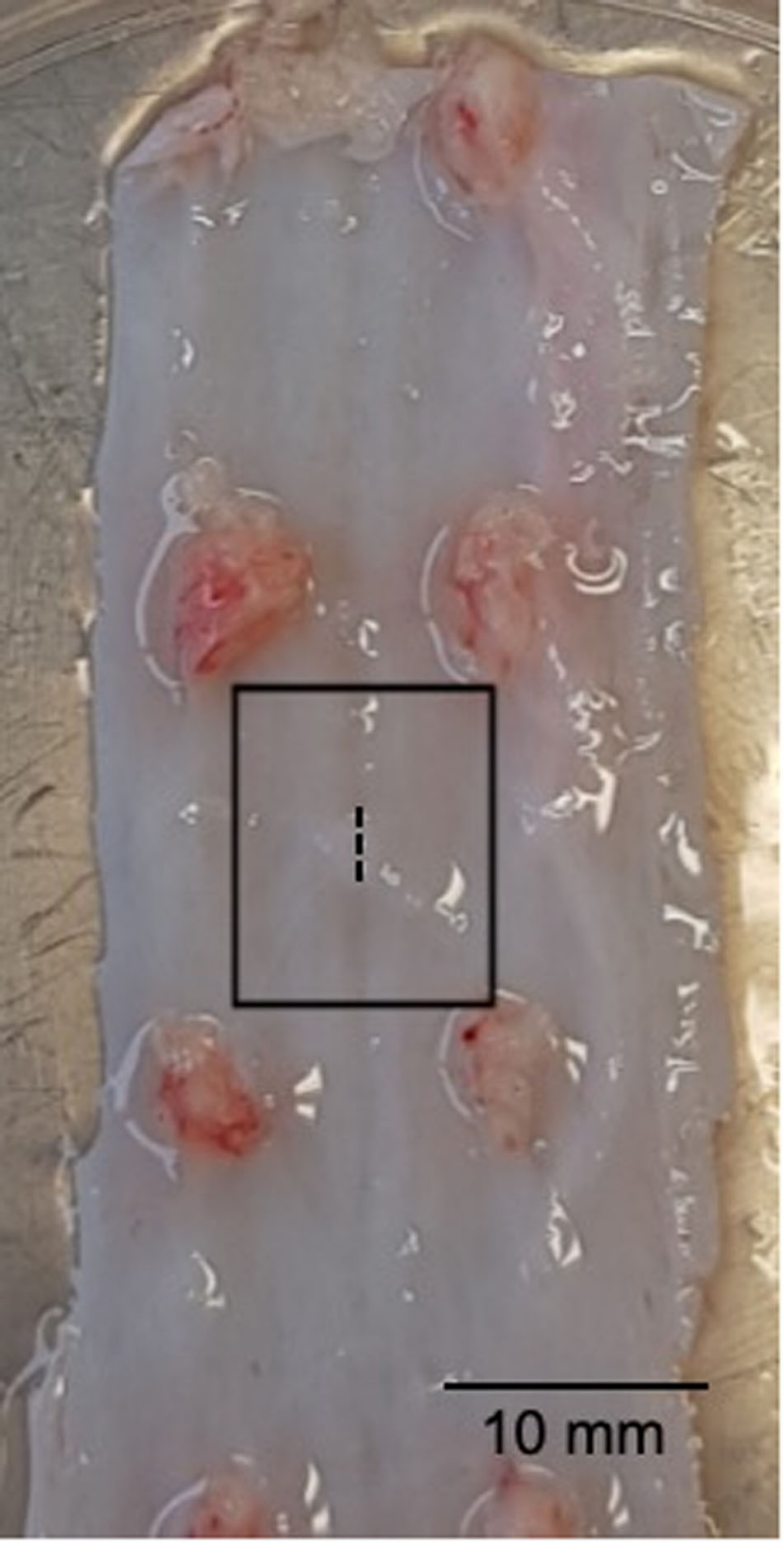
Thoracic spinal dura mater. The dotted line represents the line at which the dura is cut. The outlined rectangle indicates the test dura section

Spinal cord with intact dura mater of Dutch Landrace pigs was harvested from the abattoir. Only dura from the thoracolumbar level was used because the distance between the nerve roots of the cervical dura is too short and does not fit the designed model. The spinal dura was prepared through cutting the dura open along the spinal anterior artery to expose the dorsal aspect. In contrast to the cranial setup, the dura was not cut in a circular fashion but in a rectangular shape to fit the spinal model. Subsequently, a 3-mm midline incision was made in a cranial–caudal direction to simulate the approximate distance between two interrupted sutures in spinal dural closure. To ensure a 5-mm overlap at each side of the dura, the sealant was cut out in sizes of 13 × 10 mm (Fig. 4). For patch sealants, a weight of 500 g on a polytetrafluorethylene (PTFE) cast was used to compress the sealant on the dura. This cast guaranteed that a standardized force was exerted on the sealant. The weight was determined in the same way as done in the cranial model by letting compress a weight scale by 3 neurosurgeons. The experiments were started 1 minute after removal of the weight. For liquid sealants, sealant was applied in a hollow PTFE mould in a rectangular shape with a surface of 13 × 10 mm. Sealant was applied until the durotomy site was covered with a thickness of 1.5 mm. The PTFE mould was removed 2 min after application of the liquid sealant. For the spinal model, the cranial setup was modified for spinal conditions (Supplementary Material 2A). The plate on top of the pressure chamber of the cranial setup was replaced with a plate that followed the same curvature of human spinal dura (Supplementary Material 2B) [27]. The dura was clamped in a watertight fashion on this plate before a sealant was applied. Liqoseal, Adherus, Duraseal, Tachosil, and Tisseel Fibrin Sealant (Baxter International, Illinois, USA) were tested (Supplementary Material 2C). Tisseel was included in the spinal model as it is the most commonly used dural sealant in spinal surgery [2]. Tisseel is a hemostatic liquid glue composed of fibrinogen and thrombin that mimics blood clotting [28].

#### Spinal resistance test

For the resistance test, the cranial setup was adapted to spinal conditions. First, the plate on the top of the pressure chamber was replaced with a curved plate, as described in the acute burst pressure model. Second, the pressure in the spinal resistance test was set between 50 and 65 mmHg, in accordance with reported lumbar intraspinal pressures measured in a sitting position [29]. A speaker used in the spinal resistance setup generated a waveform that mimicked the physiologic intraspinal pressure, which is similar to the intracranial pressure waveform [30–32]. The sealant was applied as described in the acute burst pressure model. Additionally, if the sealant successfully withstood the resistance test for 72 h, a final acute pressure experiment was undertaken to test the burst pressure of the sealant after prolonged pressure. Only sealants that showed mean burst pressure results in the acute pressure test above the physiological intraspinal pressure (65 mmHg) were subjected to the resistance test.

### Statistical analysis

The cranial and spinal acute burst pressure results of Liqoseal were compared with burst pressure results of clinically used dural sealants in the same model. The ex vivo results of clinically used sealants in the cranial model have previously been published [10]. For sample size determination, an online sample size calculator was used (https://www.stat.ubc.ca/~rollin/stats/ssize/n2.html). For the cranial model, power analysis with a standard deviation of 10 mmHg, an alpha of 5%, and power at 90% yielded a minimal sample size of nine tests per sealant [10]. For the spinal model, the sample size was determined based on the cranial results, resulting in a minimal sample size of 14 tests per sealant. Finally, 20 tests per sealant were performed in the spinal model to increase the statistical precision of the results. For the resistance tests, a standard deviation of 24 h was estimated. With an alpha of 5% and power at 90%, we calculated a minimum sample size of three per group, as determined in a previous study [10]. An analysis of variance (ANOVA) was used to compare mean burst pressures of the sealants. Post-hoc Bonferroni correction was performed to adjust for multiple comparisons. A *P* value set at <0.05 was used as the threshold for statistical significance.

## Results

### Part I: the ex vivo cranial model

**Fig. 5:**
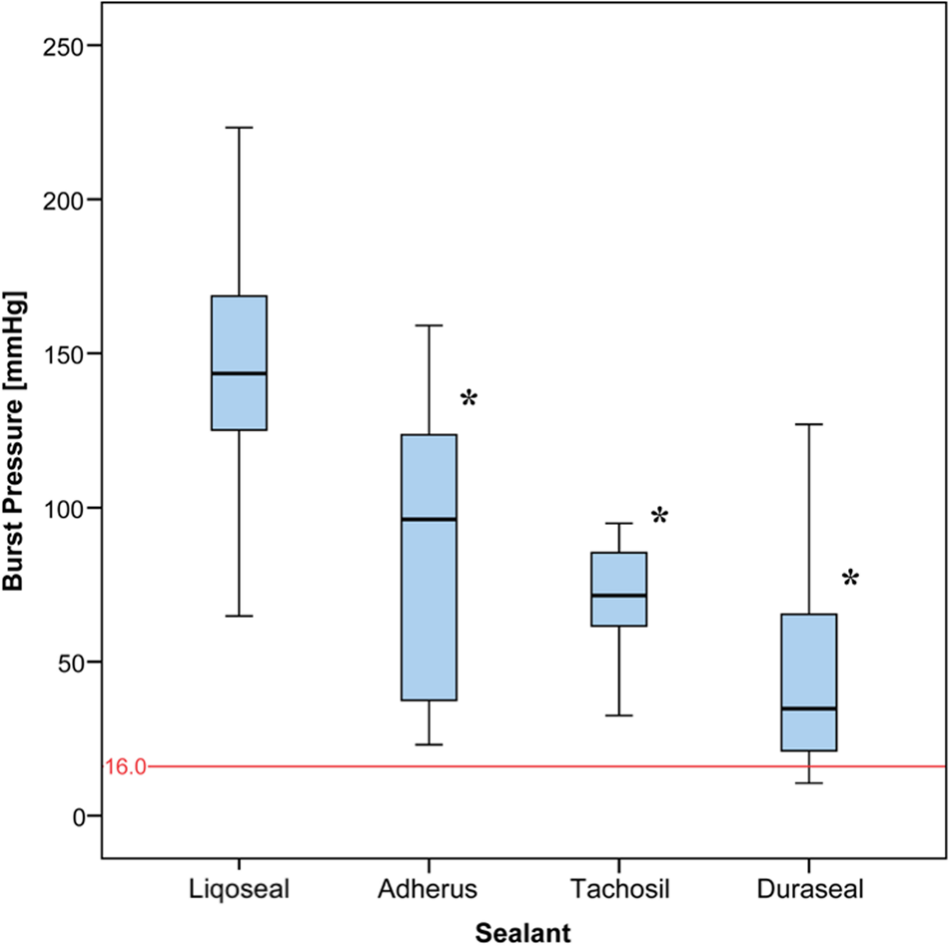
Mean acute burst pressure test results concerning the sealants used in the cranial model. The red line indicates the mean physiological intracranial pressure. * indicates significant difference compared with Liqoseal

The median time interval between harvesting and use of the dura was 1 day (range, 0–1 day). The mean dural thickness at the time of testing was 0.53 ± 0.21 mm. No significant difference was found between the groups regarding time of use and dural thickness. All the tests were performed successfully. The mean burst pressure for Liqoseal of three different batches was 145 ± 39 mmHg (165 ± 31 *n* = 20, 154 ± 28 *n* = 19 and 117 ± 40 *n* = 20), which was higher than Adherus (87 ± 47 mmHg, *n* = 14), Duraseal (51 ± 42 mmHg, *n* = 14), and Tachosil (71 ± 16 mmHg, *n* = 14) (Fig. 5). The adjusted mean differences in burst pressure between these sealants showed a significantly higher mean burst pressure for Liqoseal compared with the other sealants (Table 1).

**Table 1:** Differences in mean burst pressure between sealants in the cranial setup adjusted for multiple comparisons with post-hoc Bonferroni correction

(I) Sealant	(J) Sealant	Mean difference (I–J)	Std. error	Sig.	95% confidence interval	
					Lower bound	Upper bound
Liqoseal	Adherus	58.684*	11.377	0.000	28.04	89.33
	Tachosil	74.055*	11.377	0.000	43.41	104.7
	Duraseal	94.823*	11.377	0.000	64.18	125.47
Adherus	Liqoseal	−58.684*	11.377	0.000	−89.33	−28.04
	Tachosil	15.371	14.464	1.000	−23.59	54.33
	Duraseal	36.139	14.464	0.085	−2.82	75.1
Tachosil	Liqoseal	−74.055*	11.377	0.000	−104.7	−43.41
	Adherus	−15.371	14.464	1.000	−54.33	23.59
	Duraseal	20.768	14.464	0.926	−18.19	59.73
Duraseal	Liqoseal	−94.823*	11.377	0.000	−125.47	−64.18
	Adherus	−36.139	14.464	0.085	−75.1	2.82
	Tachosil	−20.768	14.464	0.926	−59.73	18.19

*p < 0.05

Two of three (67%) Liqoseal samples maintained attached during the 72 h resistance test in physiological conditions. One Liqoseal sample detached from the dura after 5 h. Adherus and Duraseal maintained full sealing capacities for 72 h in all tests (3 of 3). None (0 of 3) of the Tachosil sealants maintained attachment for 72 h. Tachosil released after a mean time of 1.4 h (95% confidence interval [CI] −1.8 to 4.7 h). No significant difference was found in success rates between Liqoseal, Adherus, and Duraseal.

### Part II: the ex vivo spinal model

**Fig. 6:**
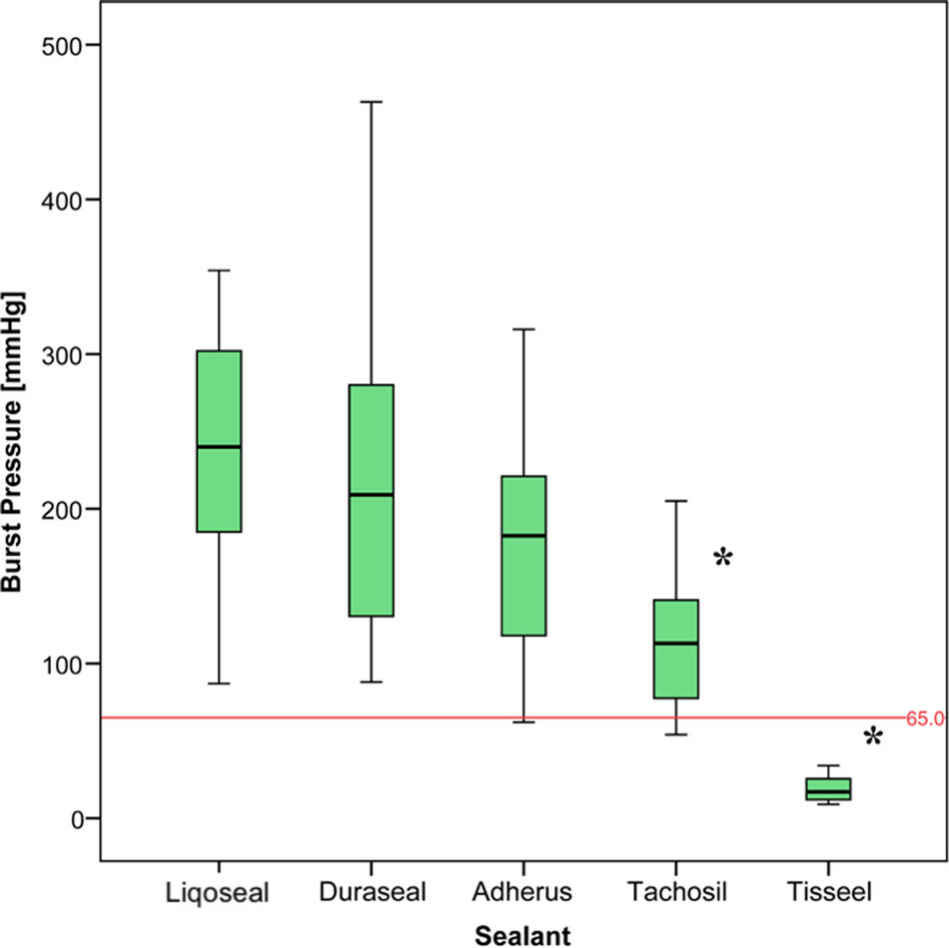
Mean acute burst pressure test results concerning the sealants used in the spinal model. The red line indicates the mean physiological intracranial pressure. Asterisk indicates significant difference compared with Liqoseal

All experiments were performed within 12 h after harvesting of the dura. The mean thickness of the dura was 0.21 mm (range, 0.15–0.25 mm). There was no significant difference in time of use and dural thickness between the groups. The mean burst pressure of Liqoseal was 233 ± 81 mmHg (*n* = 20) compared with Adherus (179 ± 71 mmHg, *n* = 20), Duraseal (216 ± 95 mmHg, *n* = 20), Tachosil (123 ± 63 mmHg, *n* = 20), and Tisseel (23 ± 16 mmHg, *n* = 20) (Fig. 6). The adjusted mean differences in burst pressure between these sealants showed a significantly higher mean burst pressure for Liqoseal compared with Tachosil and Tisseel, but not significantly higher than Adherus and Tachosil (Table 2). None of the experiments with Liqoseal and Duraseal had a burst pressure below the physiological intraspinal pressure (65 mmHg).

**Table 2:** Differences in mean burst pressure between sealants in the spinal setup adjusted for multiple comparisons with post-hoc Bonferroni correction

(I) Sealant	(J) Sealant	Mean difference (I–J)	Std. error	Sig.	95% confidence interval	
					Lower bound	Upper bound
Liqoseal	Tachosil	106.588*	24.263	0.000	36.61	176.56
	Duraseal	9.588	24.263	1.000	−60.39	79.56
	Tisseel	212.526*	23.335	0.000	145.23	279.83
	Adherus	48.647	24.263	0.482	−21.33	118.62
Tachosil	Liqoseal	−106.588*	24.263	0.000	−176.56	−36.61
	Duraseal	−97.000*	24.263	0.001	−166.97	−27.03
	Tisseel	105.938*	23.335	0.000	38.64	173.24
	Adherus	−57.941	24.263	0.192	−127.92	12.03
Duraseal	Tachosil	97.000*	24.263	0.001	27.03	166.97
	Liqoseal	−9.588	24.263	1.000	−79.56	60.39
	Tisseel	202.938*	23.335	0.000	135.64	270.24
	Adherus	39.059	24.263	1.000	−30.92	109.03
Tisseel	Tachosil	−105.938*	23.335	0.000	−173.24	−38.64
	Liqoseal	−212.526*	23.335	0.000	−279.83	−145.23
	Duraseal	−202.938*	23.335	0.000	−270.24	−135.64
	Adherus	−163.879*	23.335	0.000	−231.18	−96.58
Adherus	Tachosil	57.941	24.263	0.192	−12.03	127.92
	Liqoseal	−48.647	24.263	0.482	−118.62	21.33
	Duraseal	−39.059	24.263	1.000	−109.03	30.92
	Tisseel	163.879*	23.335	0.000	96.58	231.18

*p < 0.05

Tisseel was excluded from the chronic resistance tests as the mean burst pressure was below the physiological intraspinal pressure. Three resistance tests per sealant were performed. The mean thickness of the dura was 0.21 ± 0.02 mm and no difference in dural thickness was found between the groups. The results are shown in Table 3. One Liqoseal sample failed after 65.8 h, in which leakage occurred with a gradual decrease of pressure. In all other failed cases, a sudden loss of pressure was noted indicating bursting of the sealant. No significant difference in success rates was observed between Liqoseal and the clinically used sealants. Five acute pressure experiments were performed after 72 h. The mean acute burst pressures after 72 h for Liqoseal and Adherus remained above the physiological intraspinal pressure.

**Table 3:** Spinal resistance test results per sealant (*N* = 3)

Sealant	Duration until failure (hours)	Acute pressure after 72 h (mmHg)
Liqoseal	72, 72, 65.8	148 and 120 mmHg
Duraseal	25.7, 52.1, 64.2	NA
Adherus	72, 72, 72	170, 108, and 251 mmHg
Tachosil	0.02, 0.12, 10.4	NA

## Discussion

Systematic reviews have shown that CSF leakage still occurs after intradural surgery and that the CSF leakage rate does not decrease with sealant use. However, sealants are assumed to be an important adjunct to create a watertight closure of the dura [33]. Although CSF leakage will most likely never be eliminated in clinical practice, if a durable watertight closure could be achieved, a reduction in the CSF leakage rate would certainly be expected. In this study, we outline the design and ex vivo tests concerning a new sealant, Liqoseal. Liqoseal is the final product of an extensive development process that involved extensive reflection, experimentation, and failures. Initially, PU and DL-PLCL polymers were individually considered as biomaterial with which to develop a dural sealant patch. Two types of foam were designed simultaneously from PU and DL-PLCL. PEG-NHS was embedded in the PU and DL-PLCL foams, which yielded a homogenous foam with adhesive properties. We applied both foams to porcine dura and observed that the PU foam was too elastic and retracted itself from the dura. In contrast, DL-PLCL foam was pliable and adhered well to the dura; however, despite remaining attached, it was not watertight and artificial CSF was seeping through this foam was noted. Subsequently, we introduced a watertight layer of PU on top of the DL-PLCL foam. Combining the biomaterials resulted in the first useful prototype. Polymers other than DL-PLCL and PU were also considered; however, biodegradation, biocompatibility, or usability issues resulted in their not being tested or in their withdrawal in the early experimental stage.

Liqoseal was evaluated using the same ex vivo cranial and spinal setups as the most clinically used dural sealants were tested. The mean acute burst pressure of Liqoseal far exceeded physiological intradural pressures and was significantly higher than all the other clinically used sealants in both models, apart from Adherus and Duraseal in the spinal model. The 72 h resistance test results showed no significant difference in success rates between Liqoseal and the other clinically used sealants. Based on the overall ex vivo results, we can conclude that Liqoseal has a strong probability of preventing leakage compared to the other clinically used sealants. Moreover, Liqoseal has other potential benefits as it is comprised of completely synthetic biomaterials and, being a patch, it requires minimal preparation time, ensures equal thickness, and has consistent adhesion quality. In the cranial model, the number of sealants tested using Liqoseal was *n* = 59, because three different batches were tested. All three batches were included to avoid selection bias. The inclusion of three different batches caused a relatively high overall standard deviation. The Liqoseal design and processes will further be based on the two batches with the highest burst pressure. The high standard deviation for the liquid sealants (Duraseal and Adherus) was likely to have occurred as liquid sealants can never be applied exactly in the same way.

We noticed that in vitro studies evaluating dural sealants are rare. One prior study of Chauvet et al. have assessed the effectiveness of clinically used sealants in an in vitro dura model [6]. In their study, Duraseal and Tachosil showed highest burst pressure results. However, their study could not be compared with our study since the models differed significantly from each other. For example, they made a larger incision of 50 mm in the dura and subsequently sutured the dura, while we made only a defect of 3 mm. Our model seems to be more standardized to compare sealant with each other. The acute burst pressure results in this study were noticeably higher for the spinal model compared with the acute burst pressure results in the cranial model, regardless of the sealant being tested. The difference in burst pressure results was likely to have occurred due to differences in durotomy size and shape. In the spinal model, a 3-mm longitudinal durotomy was performed, whereas in the cranial model, a circular gap of 3 mm was punched out of the dura. One previous study also showed that the seal characteristics of sealants reduces significantly with larger defects [34]. Other possibilities to explain these differences could be the curved surface in the spinal model and the remarkably thicker cranial dura compared with the spinal dura. These setup differences may also have caused differences in resistance test results.

An unexpected finding was that, although Duraseal and Adherus showed exactly the same resistance test results in the cranial resistance test (100% success rate), no Duraseal sample (0 of 3) maintained attachment for 72 h in the spinal resistance test, whereas all Adherus samples (3 of 3) maintained attachment. It is possible that Duraseal tends to fail in spinal conditions involving a curved surface and higher setup pressure, or that a gelatinous microbial film that developed on the dura after 2 days during the resistance tests may have compromised the attachment of the sealant to the dura. This may explain why 2 of 3 Duraseal samples failed after 48 h in the spinal resistance test and why one Liqoseal sample detached after 66 h in the spinal resistance test.

This current study had several limitations. First, this study could never fully represent actual physiological conditions. For example, the effect of counter-pressure was not considered in these setups; theoretically, a replaced bone flap could substantially reinforce adhesion of the sealant to the dura. Second, no literature or instructions for use of sealant was available explaining the weight to compress a sealant. Our method of determining the ideal force was subjective and potentially not fully representing surgical reality with variances. However, in this protocol standardization was necessary. Third, autolysis and bacterial colonization of the dura may have distorted the sealant adhesion, especially in the resistance test due to the prolonged (72 h) experiment duration. We minimized these issues through harvesting the dura samples on the test day and using Antibiotic Antimycotic Solution (Sigma-Aldrich, Saint Louis, Missouri, United States) in the test setup. Finally, there was a human source of variability in sealant application. The application of the sealant is a crucial step for the durability of the adhesion. Variance in application may have influenced the outcome of the tests, especially concerning application of the liquid sealants, which may show variance in terms of the mixture of different components and in applied thickness. We attempted to eliminate this variance through preparing the sealants exactly according to the instructions of use, and through limiting the number of experiment performers (SR and SvT, both of whom had extensive training) and using molds for all tests.

The biocompatibility and biodegradability of the biomaterials used to develop Liqoseal, consisting of PU, DL-PLCL, and PEG-NHS, have already been evaluated [11–23]. These biomaterials showed no biocompatibility and biodegradability issues. However, the composition, molecular structure (number of arms, arm length, molecular weight) and concentration of these biomaterials as Liqoseal consisted of, are not evaluated yet. Therefore, one of the most important next steps after this study is testing the biocompatibility and biodegradability of Liqoseal in an in vivo model. A subsequent clinical trial should study the safety and efficacy of Liqoseal in a randomized controlled trail comparing the device with the current standard of care.

## Conclusion

Liqoseal is capable of achieving a strong watertight seal of a dural defect in conditions simulating cranial and spinal application on the dura. Liqoseal was shown to have a high probability of preventing CSF leakage compared with current clinically used sealants. In vivo studies are necessary to evaluate the safety, biocompatibility, and biodegradability of Liqoseal. Subsequently, clinical trials should aim to demonstrate the effectiveness of Liqoseal in preventing CSF leakage.

## Supplementary information

Supplementary Figure 1A

Supplementary Figure 1B

Supplementary Figure 1C

Supplementary file legends
