# Supplementary figures and images for: Ex vivo evaluation of a multilayered sealant patch for watertight dural closure: cranial and spinal models
Source: J Mater Sci Mater Med. 2021 Jul 23;32(8):85. doi: 10.1007/s10856-021-06552-4 (PMC8302489; doi:10.1007/s10856-021-06552-4)

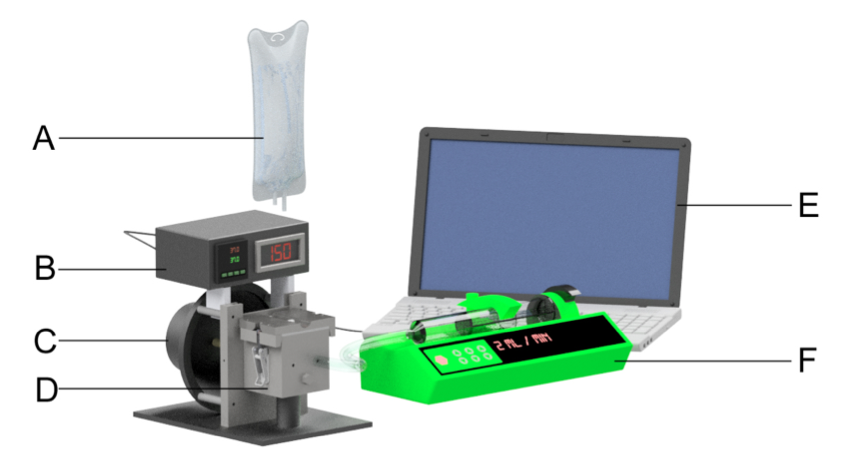

Supplement: Supplementary file 1 — Supplementary Figure 1A [file 10856_2021_6552_MOESM1_ESM.tif]

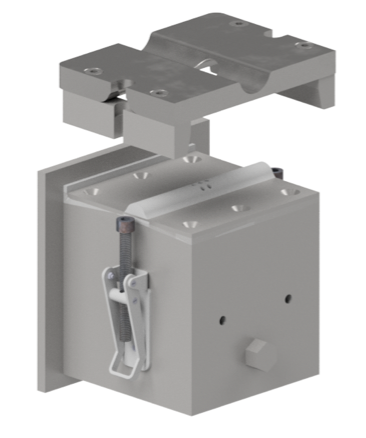

Supplement: Supplementary file 2 — Supplementary Figure 1B [file 10856_2021_6552_MOESM2_ESM.tif]

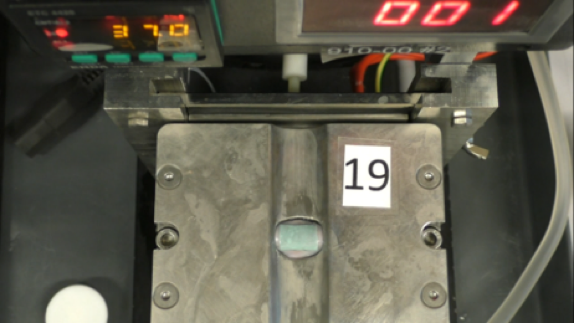

Supplement: Supplementary file 3 — Supplementary Figure 1C [file 10856_2021_6552_MOESM3_ESM.tif]
