# Supplementary material for: Ex vivo evaluation of a multilayered sealant patch for watertight dural closure: cranial and spinal models
Source: J Mater Sci Mater Med. 2021 Jul 23;32(8):85. doi: 10.1007/s10856-021-06552-4 (PMC8302489; doi:10.1007/s10856-021-06552-4)
Supplement: Supplementary file 4 — Supplementary file legends [file 10856_2021_6552_MOESM4_ESM.docx]

**Supplementary file legends**

**Figure 1A.** *Ex vivo* spinal acute burst pressure and resistance setup. A) Dripper, filled with artificial CSF to moisten the dura and the sealant. B) Pressure meter and thermometer. C) Speaker, generating pressure waves. D) Container, filled with artificial CSF onto which the dura was clamped in a watertight fashion. E) Computer, calculating the pressure curve. F) Fluid pump, generating pressure in the container.

**Figure 1B.** Spinal pressure chamber consisting of a bottom plate with the same curvature as human spinal dura and an upper plate to clamp the dura between the two plates.

**Figure 1C.** Top view of the setup in which the dura with Liqoseal has been clamped in a watertight fashion.
